# Top-down effects of a lytic bacteriophage and protozoa on bacteria in aqueous and biofilm phases

**DOI:** 10.1002/ece3.1302

**Published:** 2014-11-10

**Authors:** Ji Zhang, Anni-Maria Örmälä-Odegrip, Johanna Mappes, Jouni Laakso

**Affiliations:** 1 Centre of Excellence in Biological Interactions, Department of Biological and Environmental Science, University of JyväskyläP.O. Box 35, 40014, Jyväskylä, Finland; 2 Department of Biosciences, University of HelsinkiP.O. Box 65, 00014, Helsinki, Finland

**Keywords:** *Acanthamoeba castellanii*, aquatic bacteria, defense evolution, lytic bacteriophage, microcosm, Semad11, *Serratia marcescens*Db11, *Tetrahymena thermophila*, top-down regulation

## Abstract

Lytic bacteriophages and protozoan predators are the major causes of bacterial mortality in natural microbial communities, which also makes them potential candidates for biological control of bacterial pathogens. However, little is known about the relative impact of bacteriophages and protozoa on the dynamics of bacterial biomass in aqueous and biofilm phases. Here, we studied the temporal and spatial dynamics of bacterial biomass in a microcosm experiment where opportunistic pathogenic bacteria *Serratia marcescens* was exposed to particle-feeding ciliates, surface-feeding amoebas, and lytic bacteriophages for 8 weeks, ca. 1300 generations. We found that ciliates were the most efficient enemy type in reducing bacterial biomass in the open water, but least efficient in reducing the biofilm biomass. Biofilm was rather resistant against bacterivores, but amoebae had a significant long-term negative effect on bacterial biomass both in the open-water phase and biofilm. Bacteriophages had only a minor long-term effect on bacterial biomass in open-water and biofilm phases. However, separate short-term experiments with the ancestral bacteriophages and bacteria revealed that bacteriophages crash the bacterial biomass dramatically in the open-water phase within the first 24 h. Thereafter, the bacteria evolve phage-resistance that largely prevents top-down effects. The combination of all three enemy types was most effective in reducing biofilm biomass, whereas in the open-water phase the ciliates dominated the trophic effects. Our results highlight the importance of enemy feeding mode on determining the spatial distribution and abundance of bacterial biomass. Moreover, the enemy type can be crucially important predictor of whether the rapid defense evolution can significantly affect top-down regulation of bacteria.

## Introduction

Bacterial communities are regulated by the availability of resources (“bottom-up control”) and by organisms at higher trophic levels (“top-down control”) (Pace and Cole 1994). Bacteriophages and protozoa are the two major biotic causes of bacterial mortality, although at a given time point and place, one or the other enemy group may dominate (Fuhrman 1999; Suttle 2005). This makes bacteriophages and protozoa potential candidates for biological control of bacterial pathogens (Goodridge and Bisha 2011; Ali and Saleh 2013). However, little is known about the relative impacts of bacteriophages and protozoa on the bacterial communities in aqueous and biofilm phases. There are many studies on the relative impacts of protozoan predators and bacteriophages in regulating bacterial abundance, but results of these studies are versatile: for example, estimates of virus-induced mortality in natural aquatic systems vary from 20% to 100% (Bettarel et al. 2004). The reason for this could be that most of the data are collected from the field (the estimated biomass consumption is often confounded by many other environmental factors, such as temperature, resource availability, and light) (Mathias et al. 1995; Wommack et al. 1996), and the prey effect size is often measured indirectly (Mcmanus and Fuhrman 1986; Sherr et al. 1986; Nygaard and Hessen 1990). Thus, the impact of bacteriophages and protozoa in controlling bacterial biomass should also be studied by directly measuring the biomass changes in controlled experimental settings.

We conducted a microcosm experiment where opportunistic bacterial pathogen *Serratia marcescens* was exposed to lytic bacteriophage Semad11 and to two typical protozoan predators: particle-feeding ciliate *Tetrahymena thermophila* and surface-feeding amoeba *Acanthamoeba castellanii*. To study the impact of protozoa and bacteriophages on the temporal and spatial dynamics of bacterial biomass in aqueous and biofilm phases, the bacteria were cultured either alone, with single enemy or with all the enemies of the enemies in static microcosms, simulating freshwater pond environment, for 8 weeks (approximately 1300 bacterial generations), during which the bacterial biomass in the free-water phase and biofilm phase were measured weekly. Also, the population dynamics of the protozoan predators was followed throughout the experiment. There are many advantages in such experimental settings, including direct measurement of the bacterial biomass, easy manipulation of environmental variables such as nutrition, temperature, and light, possibilities to store and revive the experimental strains that allow direct comparison of ancestral and evolved strains. Such microcosm experiments also allow large population sizes and number of generations (Elena and Lenski 2003).

We hypothesize that: (1) parasitic bacteriophages are initially highly effective in regulating bacterial abundance, whereas the effect of protozoans increases more slowly due to the differences in generation time; (2) The differing feeding modes of the protozoans can create spatial differences in the bacterial community structure: As a surface feeder, the amoeba was expected to reduce predominantly the bacterial biofilm, whereas particle-feeding ciliates reduce the open-water biomass; (3) The evolution of bacteriophage-resistance is predicted to rapidly diminish the bacteriophage effect on bacteria (Buckling and Rainey 2002; Abedon 2008; Gomez and Buckling 2011), but see: Bohannan and Lenski (2000), Morgan et al. (2009)); (4) Theory and previous experimental work on bacteria–ciliate system suggests that the ability of protozoan predators to coevolve is limited compared to the bacterial prey (Friman et al. 2008). Thus, we expected that the bacterial prey defense evolves stronger and the biomass regulation by protozoans will slowly diminish in time; and (5) The combined effects of all enemy groups on the bacteria were expected to be most pronounced because that leaves the bacteria least opportunities for spatial refuges and also prevents more effectively the defense evolution if the defenses are costly and group specific.

Our results suggest that the feeding mode of the enemy affects the spatial distribution and abundance of bacteria. Particle-feeding ciliates were the most efficient enemy type in reducing bacterial biomass in the open-water, but least efficient in reducing the biomass in the biofilm, whereas amoebae reduced both bacteria in the open-water phase in biofilm. The effect of amoeba grazing on the bacterial biofilm was most pronounced toward the end of the experiment. The long-term negative effect of bacteriophages on bacterial biomass was almost negligible, although experiments with the ancestral bacteriophages and bacteria showed that bacteriophages had the ability to lyse most of the bacterial biomass in the open-water phase within 24 h. This shows that the effect of top-down regulation by bacteriophages is time-dependent and minor in the long run due to rapid defense evolution, in comparison with top-down regulation by protozoans. The combined effect of the enemies was most pronounced for biofilm biomass.

## Materials and Methods

### The study species and strains

Bacteria *S. marcescens* strain Db11 (Flyg et al. 1980) was kindly provided by Prof. Hinrich Schulenburg, and it was initially isolated from dead *Drosophila* fruit fly. *S. marcescens* is an environmentally growing gram-negative bacterium, that is, also opportunistically pathogenic infecting a broad spectrum of hosts, including plants, corals, nematodes, insects, fish, and mammals (Grimont and Grimont 1978; Flyg et al. 1980). *S. marcescens* is commonly present as a free-living form in soil, freshwater, and marine ecosystems (Sutherland et al. 2010; Mahlen 2011), and frequently encounters parasitic and predatory enemies.

The predatory particle-feeding ciliate *Tetrahymena thermophila* strain ATCC 30008 (minimum generation time about 2 h (Kiy and Tiedtke 1992)) was obtained from American Type Culture Collection and is routinely maintained in PPY (proteose peptone yeast medium) at 25°C (Friman et al. 2008).

Free-living amoeba *Acanthamoeba castellanii* strain CCAP 1501/10 (generation time about 7 h (Kennedy et al. 2012) was obtained from Culture Collection of Algae and Protozoa (Freshwater Biological Association, The Ferry House, Ambleside, United Kingdom) and routinely maintained in PPG (proteose peptone glucose medium [Page 1976]) at 25°C.

**Figure 1:**
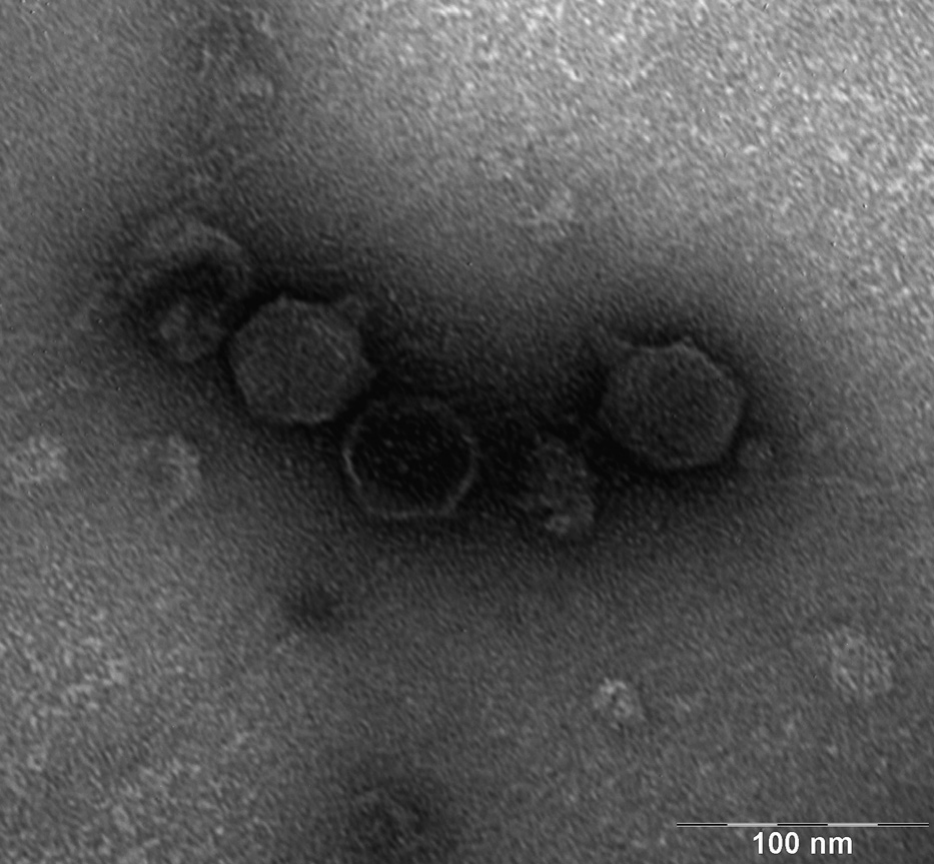
Bacteriophage Semad11.

Obligatory lytic bacteriophage Semad11 infecting *S. marcescens* Db11 was isolated from a sewage treatment plant in Jyväskylä, Finland in 2009. Semad11 is a T7-like bacteriophage belonging to *Podoviridae* (A-M. Örmälä-Odegrip, unpubl. data) (Fig. 1).

### Long-term coculture experiment

NAS (New Cereal Leaf – Page's modified Neff's amoebae saline) medium used in the long-term experiment was prepared as follows: 1 g of cereal grass powder (Aldon Corp., Avon, NY) was boiled in 1 liter of dH_2_O for 5 minutes and then filtered through a glass fiber filter (GF/C, Whatman). After cooling down, 5 ml of PAS stock solution II and I was added and restored the final volume with deionized water to 1 liter (Page 1988; La Scola et al. 2001; Greub and Raoult 2004).

Before the experiment, the organisms were cultured separately and prepared as follows. For bacterial culture, a single colony of *S. marcescens* strain Db11 was seeded to 80 mL of NAS medium in a polycarbonate Erlenmeyer flask capped with membrane filter (corning). The flask was incubated at 25°C on rotating shaker (120 rpm) for 48 h.

The amoeba and ciliate cells were harvested and washed twice in 40 mL of PAS (Page's amoeba saline) with centrifugation at 1200 × g for 15 min to pellet the cells. After the centrifugation, cells were suspended in PAS and adjusted to final concentration of ca. 10 cells *μ* L^−1^.

To prepare the bacteriophage stock, LB-soft agar (0.7%) from semi-confluent plates was collected and mixed with LB (4 mL per plate) and incubated for 3.5 h at 37°C. Debris was removed by centrifugation for 20 min at 9682 × g at 5°C. Stock was filtered with 0.2  *μ* m Acrodisc® Syringe Filters (Pall). The bacteriophage stock was diluted 1:100 000 in NAS medium, giving approximately 10^6^ PFU mL^−1^.

The long-term experiment was initiated in 25 cm^2^ polystyrene flasks with nonwettable 0.2- *μ* m hydrophobic filter membrane caps (Sarstedt TC 83.1810.002). Each flask was inoculated with 1 mL of appropriate microorganism (s) depending on the community composition, and the total volume was adjusted to 15 mL with NAS medium. There were five community composition treatments: (1) bacteria; (2) bacteria + ciliate; (3) bacteria + amoeba; (4) bacteria + bacteriophage; and (5) bacteria + all three enemies. Each treatment was replicated in 36 flasks.

The static liquid cultures were incubated at 25°C. Every 7 days, the contents of each flask were mixed thoroughly, and 50% of the volume was replaced with fresh NAS medium, making the system a pulsed resource type (Friman and Laakso 2011; Friman et al. 2011). Before every renewal, four flasks from each treatment were randomly chosen for destructive sampling.

### Measurement of bacterial biomass and protozoan population size

Bacterial biomass in the free-water phase was measured from five separate 400  *μ* L samples from each flask, on honeycomb 2 plates (Oy Growth Curves Ab Ltd Helsinki, Finland). The amount of biomass was measured as optical density (OD) at 460-580 nm wavelength using Bioscreen C® spectrophotometer (Oy Growth Curves Ab Ltd). The measurements were repeated 10 times at 5-min interval.

To measure the amount of *S. marcescens* biofilm attached to the walls, 15 mL of 1% crystal violet solution (Sigma-Aldrich St. Louis, Missouri, USA) was injected to the flasks. After 10 min, the flasks were rinsed with distilled water for three times, and then, 15 mL of 96% ethanol was added to flasks to dissolve crystal violet from the walls for 24 h (O'toole and Kolter 1998). The amount of formed biofilm was quantified by the absorbance of crystal violet-ethanol solution as above. The amount of formed biofilm was quantified with the OD of crystal violet-ethanol solution at 460–580 nm with Bioscreen C® spectrophotometer (Friman and Laakso 2011).

The bacteriophage population density can be measured, for example, by flow cytometry (Brussaard et al. 2000). However, the total number of bacteriophage particles in the systems is not a meaningful measure, as the bacteriophage abundance tells nothing about the infectivity of the bacteriophages against bacterial genotypes (or phenotypes) present in the system. However, we confirmed that bacteriophages were present in the microcosms throughout the experiment by taking three independent 500  *μ* L samples from each flask. To remove the host bacteria, amoebas, and ciliates, the samples were treated with chloroform and centrifuged with 17,000 × g for 7 min. Ten microliters spots of supernatant were then added to 1.5% agar plates containing an upper layer of 0.7% LB-agar mixed with 200  *μ* L of overnight grown ancestor Db11 cells, and the plates were incubated overnight in 25°C. All samples from all flasks throughout the experiment formed plaques on the bacterial lawn confirming that bacteriophages did not go extinct during the experiment.

To follow the population dynamics of the amoeba, the flasks were carefully flipped upside down, and eight randomly placed images (total area 18 mm^2^) from the microcosm bottom wall were digitized with an Olympus SZX microscope (Olympus Optical Co., Ltd. Tokyo, Japan.) (32 × magnification) in the dark-field mode. The cell numbers in each image were counted with a script developed in our lab for the Image Pro Plus software (v. 7.0) (J. Zhang, unpubl.).

To determine the ciliate density, 250  *μ* L of open-water sample was mixed with 10  *μ* L Lugol solution and then injected into a glass cuvette race (depth 2.34 mm) so that the ciliate cells can be instantly stained and fixed. For each sample, eight randomly placed images (total area 18 mm^2^) were digitized with an Olympus SZX microscope (32 ×  magnification). The cell numbers in each image were counted with an Image Pro Plus script (Laakso et al. 2003).

### Short-term experiments with bacteriophage Semad11 and bacteria Db11

Db11 was grown on an LB plate at 25°C for 48 h and a single colony was inoculated into NAS medium. Liquid culture was incubated in shaker (120 rpm) at 25°C for 48 h. Five microliters of bacterial culture was seeded into 200 wells of honeycomb 2 plates (Oy Growth Curves Ab Ltd) containing 400  *μ* L of NAS medium. Hundred wells received simultaneously 5  *μ* L of bacteriophage stock in dH_2_O with approximately 10^4^ PFUs. The OD was measured at 460–580 nm wavelength using Bioscreen C® spectrophotometer (Oy Growth Curves Ab Ltd). The measurements were repeated at 5-min intervals for 100 h. The presence of infectious Semad11 was confirmed (with ancestral Db11) from the phage-treated wells at the end of measurements.

### Statistical analysis

In addition to open water and wall biomass of bacteria and protozoa density, we calculated the ratio of open-water to biofilm biomass and total bacterial biomass in the system. These response variables were measured weekly in samplings where four flasks per treatment were selected for destructive sampling. Thus, we used ANOVA where time and community combination were treated as factors (response variable = community + time + community × time + ***ε***). When needed, the response variables were square root or log-transformed to meet the assumptions of the analysis.

Friedman's test and Wilcoxon signed-rank test was used to compare the impacts of different bacterial enemies and enemy combinations in reducing the bacterial biomass in biofilm phase and in free-water phase (Table1). All the analyses were done with SPSS v. 19 (IBM New York, NY, USA.).

**Table 1:** Wilcoxon's signed-rank test showing the efficiency of different enemy compositions in reducing bacterial growth in biofilm and in open water. Sample size in all comparisons is 4.

	Open water		Biofilm	
	*Z*	*P*	*Z*	*P*
DACP < DA	−5.121	< 0.001	−5.055	< 0.001
DC < DA	−5.121	< 0.001		
DC = DA			−1.605	0.109
DP > DA	−5.160	< 0.001		
DP = DA			−1.285	0.199
DC > DACP			−4.590	< 0.001
DC = DACP	−1.414	0.157		
DP > DACP	−5.039	< 0.001	−3.808	< 0.001
DP < DC			−3.190	< 0.001
DP > DC	−5.058	< 0.001		

## Results

### Bacterial biomass dynamics

**Figure 2:**
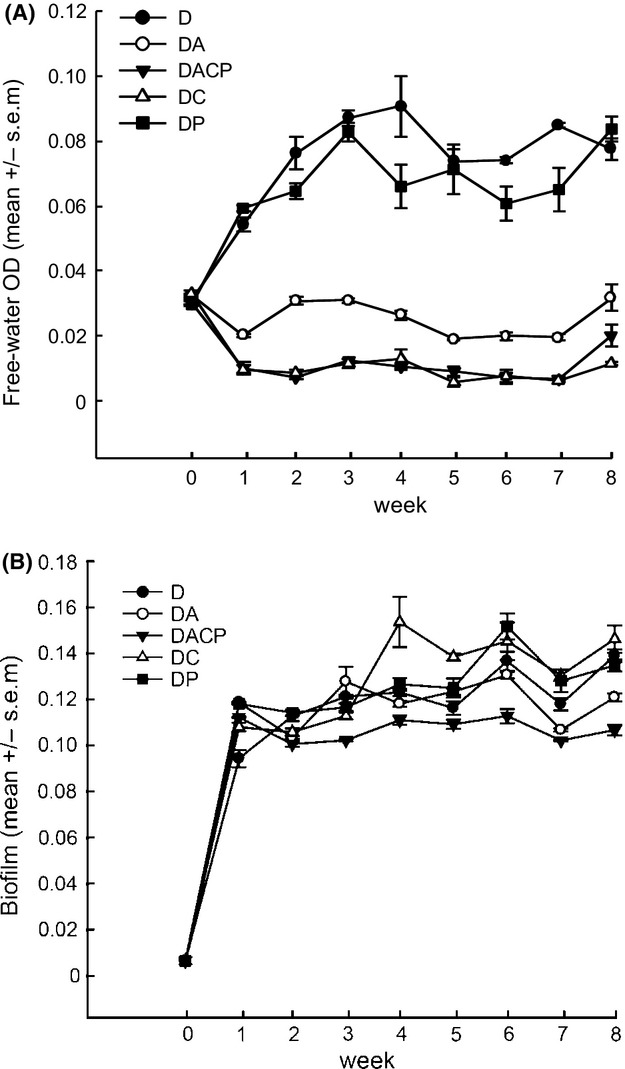
Open-water bacterial biomasses during the 8-week experiment, measured as optical density (OD) (panel A). Panel B represents amount of bacterial biofilm within different treatments. D =  *Serratia marcescens* strain Db11; A = amoeba; C = ciliate; P = phage.

The protozoans and bacteriophages had highly variable efficiencies in reducing bacterial biomass (OD) in the open-water phase and the efficiency also varied temporally (enemy: *F*_4, 159_ = 1049.63, *P*  < 0.001; time: *F*_7, 159_ = 16.37 *P*  < 0.001, time×enemy: *F*_28,159_ = 2.59, *P*  < 0.001, Fig.2A). The OD in the free-water phase was reduced most in the treatments with ciliates (on average 88% during the weeks 1–8), whereas the bacteriophage had only a minor effect (on average 9% during the weeks 1–8). The amoeba had an intermediate effect (on average 68% during the weeks 1–8). The effect of all enemies together was the same as the effect of ciliates alone.

**Figure 3:**
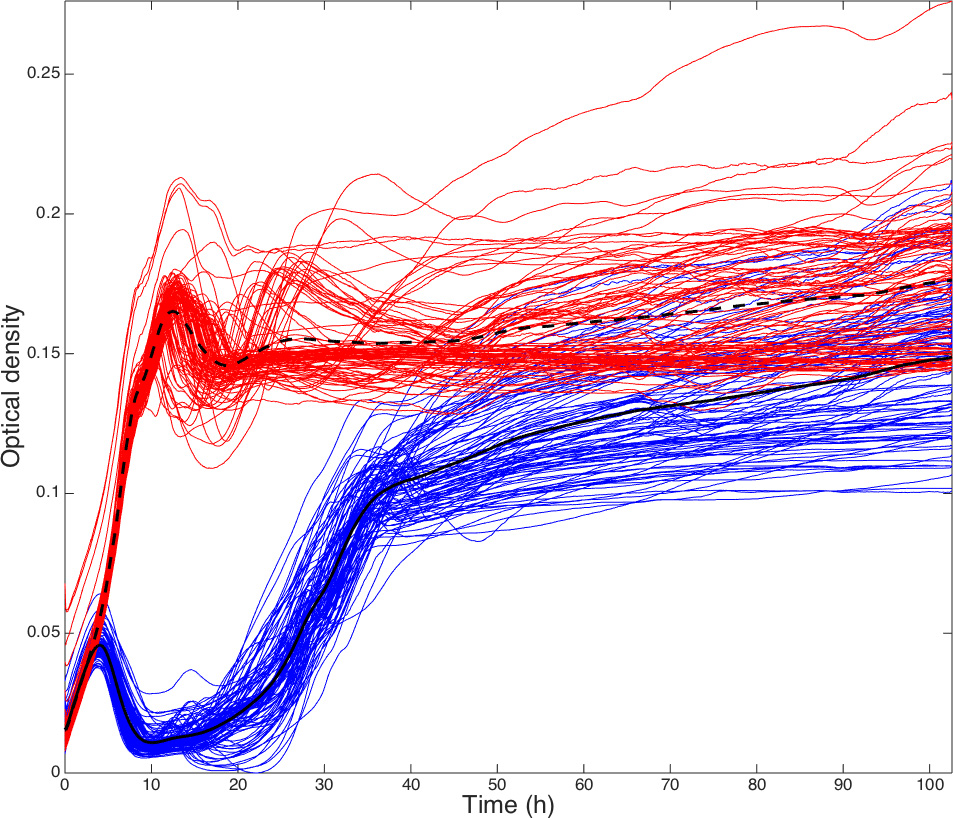
Effect of phage Semad11 on the bacterial biomass of *Serratia marcescens* strain Db11 measured as optical density (OD). Red lines show bacterial biomass dynamics without bacteriophages, and blue lines biomass dynamics with the bacteriophage. Black lines show the mean of the two groups. Bacteriophages initially crash the biomass by 93% during the first 15 h, after which the phage-resistant variants emerge. *N*  = 100 for both treatment groups.

The short-term experiment with the ancestor bacteria and the ancestor bacteriophage showed an extremely rapid evolutionary dynamics that was not captured by the one-week sampling frequency of the long-term experiment: In the short-term experiment, we found that the bacteriophage resulted in a > 93% crash of the biomass within 12 h (Fig.3). However, the bacteria developed defense against the bacteriophages within the first 24 h, and after 100 hours, the bacteriophage-exposed bacterial biomass was close to that of the bacteriophage-free systems.

The amount of attached biofilm increased rapidly during the first week (Fig.2B). Thereafter, the effect of bacterial enemies on surface attached biofilm biomass depended on the enemy identity and time (enemy: *F*_4, 159_ = 55.18, *P*  < 0.0001; time: *F*_7, 159_ = 40.68, *P*  < 0.001; time×enemy: *F*_28, 159_ = 7.60, *P*  < 0.001). The greatest long-term enemy-induced loss of the biofilm biomass was found in the treatment where all three types of enemies were present and amoebae had an intermediate negative effect on biofilm biomass during the last three weeks. The bacteriophages and ciliates had no effect, or even transiently increased biofilm biomass.

### Comparisons of bacterial enemies in reducing the bacterial biomass within open-water and biofilm

We found that different enemies and enemy combinations had a significant effect on the bacterial biomass both in the open water and in biofilm (Chi-square 116, 5, df = 4, *P*  < 0.001; Chi-square = 49, 6; df = 4, *P*  < 0.001). In the open water, ciliates were the most efficient predators in consuming bacterial biomass because they were alone as efficient as all three enemy types combined (DC = DACP, *Z*  = –1–414, *P*  = 0.157). Amoebas and phages were both alone less efficient than ciliates, but amoebas were more efficient than phages. Statistics for all comparisons are shown in the Table1.

Within the biofilm, the three enemy type combination was ranked the most efficient (all *P* -values < 0.0001). Statistics for all pair wise comparisons on biofilm reduction efficiency are shown in the Table1.

### Enemy population dynamics

**Figure 4:**
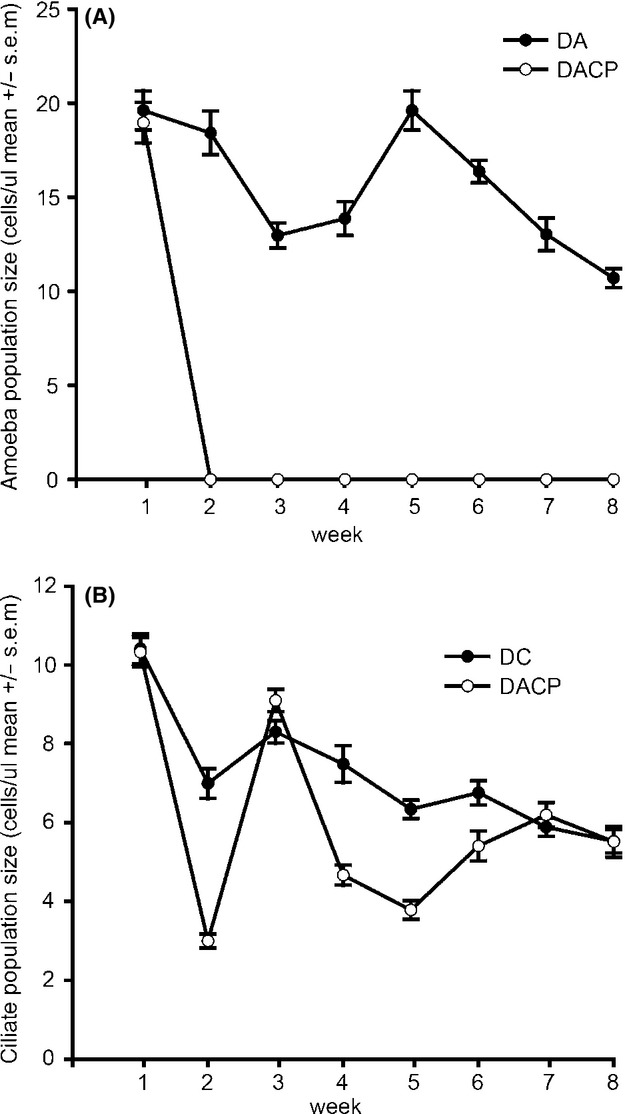
Population dynamics of the ciliate (panel A) and amoeba (panel B), within the different treatments in the 8-week experiment. The amoeba density in the multienemy treatment was likely to be underestimated after the first week (see Materials and Methods), and the amoeba population might have persisted throughout the experiment at low density or as cysts. D =  *Serratia marcescens* strain Db11; A = amoeba; C = ciliate; P = phage.

The ciliate population size was on average higher in the single-enemy treatment than in the treatment mixed with bacteriophage and amoeba (enemy *F*_1, 511_ = 57.001, *P*  < 0.001, time *F*_7, 511_ = 71.072, *P*  < 0.001, time×enemy *F*_7,511_ = 14.693, *P*  < 0.001; Fig.4A). Moreover, in the single-enemy treatment, the ciliate density declined consistently in time, whereas in the multienemy treatment population sizes seemed fluctuating more. Amoeba cells in the multienemy treatment were not visible under the dark-field microscope after the first week. They persisted in the single-enemy treatment and also declined in time (enemy *F*_1, 282_ = 561.079, *P*  < 0.001, time *F*_8,282_ = 28.687, *P*  < 0.001, time×enemy *F*_24, 282_ = 14.075, *P*  < 0.001; Fig.4B).

Bacteriophages were present in all microcosms throughout the experiment, which was verified by infecting the ancestor bacteria with the bacteriophage populations isolated from the end of the experiment (see Materials and Methods).

## Discussion

Top-down control by protozoan predators and parasitic bacteriophages is the most prominent cause of bacterial mortality in natural microbial communities (Fuhrman 1999; Jürgens and Matz 2002), and thus, phages and protozoa are considered as potential biocontrol agents against bacterial pathogens (Goodridge and Bisha 2011; Ali and Saleh 2013). However, the relative importance of these bacterial enemies is unclear due to methodological difficulties in assessing the relative mortalities and uncontrollable environmental variables. Our coculture experiment in static microcosms with protozoans (amoeba *A. castellanii*, ciliate *T. thermophila*), bacteriophages (lytic bacteriophage Semad11) and environmentally growing opportunistic pathogenic bacteria (*S. marcescens* strain Db11) demonstrated that bacterial enemies with different feeding strategies had very different spatial and temporal effects on the bacterial population.

Bacteriophages and protozoan predators use quite different strategies to utilize bacterial biomass. For example, most flagellates and ciliates are specialized in preying on suspended bacteria, whereas amoebae are thought to almost exclusively feed on biofilm (Rodriguez-Zaragoza 1994; Molmeret et al. 2005); but see Bowers (1977)). As for bacteriophages are parasites that attack bacteria in the free water as well as those hidden in biofilms (Hanlon et al. 2001). The capability of a bacteriophage to infect biofilms (formed by an otherwise susceptible bacterial host) depends on viral access on bacterial surface, which in turn is determined by the structure of biofilm and the capability of bacteriophage to degrade the extracellular polymers forming the matrix of the biofilm (Sutherland et al. 2004; LaBrie et al. 2010).

Although the assays for assessing the amount of biomass in the open water and biofilm were different, and thus not directly comparable, by comparing the ranks of the bacterial enemies within the two results, we can see that treatment with all three enemies is ranked equal with the treatment with ciliates alone. Thus, ciliates can be concluded to account for the strongest long-term negative effect in reducing bacterial biomass in the open water, but not in biofilm (Table1). Ciliates use oral groove to engulf suspended bacteria and select the prey mostly based on size (Fenchel 1980; Gonzalez et al. 1990; Christaki et al. 1998), and it has been shown that *Tetrahymena* spp. ciliates can also readily consume biofilms formed by *Pseudomonas* spp. or *Serratia* spp. (Eisenmann et al. 1998; Weitere et al. 2005) and change the composition and abundance of bacteria in the biofilm (Parry et al. 2007; Dopheide et al. 2011). However, we did not find that the bacterial biofilm biomass dynamics in the ciliate–bacteria or bacteriophage–bacteria treatments differed from bacteria cultured alone during the eight-week experiment. One explanation could be that when the ciliates feed the open-water biomass, it frees the common bacterial resources that can increase the growth of the biofilm biomass and thus compensate for the potential feeding of the biofilm biomass by ciliates. However, the lowest amount of biofilm was found in the system where ciliates, amoebae, and bacteriophages were all present. These results indicate that the effect of ciliates and bacteriophages on the amount of biofilm was of significance only when the amoebae were present (Fig.2B and Table1).

Contrary to ciliates, amoebae are thought to be specialized surface grazers (Rodriguez-Zaragoza 1994; Molmeret et al. 2005). In our experiment, the surface grazing effect was visible on biofilm biomass. However, this reduction was most pronounced during the three final weeks of our experiment (Fig2B). Notably, amoebae were also able to significantly reduce the bacterial biomass in the free water (Fig2A and Table1). One explanation is that the amoebae consumed the suspended bacteria through pinocytosis (Bowers 1977; Vogel et al. 1980). This niche overlaps with ciliates is also consistent with the observed ciliate population dynamics: The ciliate population size was on average higher in the single-enemy treatment than in the treatment mixed with bacteriophage and amoeba, and within the multienemy treatment, the ciliate densities transiently increased right after the decline of amoeba populations (Fig.4A). The amoeba decline was severe, as the cells were not detectable with the dark-field microscope after 1 week (Fig.4B). Although dark-field microscopy is a good tool to observe amoeba cells in the normal conditions (Palsson et al. 1997), it might not be sufficient to count the amoeba cells in the condition of starvation. Thus, the amoeba density was likely to be somewhat underestimated, especially in conditions where competition for food was severe, that is, the amoeba population might have persisted throughout the experiment as atypical form in the treatment with all enemies. The strong decline of amoeba also indicates that the competition between ciliates and amoeba was asymmetric in favor of the ciliates, which are known to be efficient predators of bacteria under similar microcosm conditions (Meyer and Kassen 2007; Friman et al. 2008; Friman and Buckling 2013).

In contrast to the predators, the lytic bacteriophages had the least long-term effect on bacterial density in the open water and had no effect on the amount of the attached biofilm. However, when looking at the short-term dynamics between Semad11 and Db11, bacteriophages proved to be extremely effective in killing their hosts. Our separate short-term experiments demonstrated an initial 93% decrease of the bacterial population in 12 h. High mortality risk is likely to create strong selection pressure on the host defense, resulting in bacteria becoming involved in a tight coevolutionary arms-race dynamics with bacteriophages, where the fitness of a particular host and parasite genotype depends on time (Van Valen 1973; Dawkins and Krebs 1979; Buckling and Rainey 2002; Friman et al. 2008). Dynamics corresponding to this scenario were also observed in our experiment; the initial rapid decrease of bacterial abundance was only temporary, as phage-resistant bacteria were capable of restoring the populations close to the previous size within 100 h (Fig.3). Thus, the observed appearance of phage-resistance seemed to effectively weaken the trophic link between the parasitic phages and their host bacteria.

This finding also demonstrates one of the major challenges in phage therapy – the rapid evolution of defense (Chan et al. 2013). However, the results also show, that although phage-resistant bacteria quickly emerge, the bacterial populations were never able to reach to level of the control group where the bacteria were grown alone (Fig.2A). This could be because of the emergence of a new phage genotype limiting the gross bacterial population, presumably as a result of evolutionary arms race (Gomez and Buckling 2011; Stern and Sorek 2011). Alternatively, phage-resistance could be phenotypic (e.g., masking of phage-receptors on the cell surface), (Bull et al. 2014), resulting in a trade-off with the growth ability. It has been shown, although that the use of a phage cocktail instead of a single phage delays the appearance of phage-resistant bacterial variants (Levin and Bull 2004; Gu et al. 2012), which brings about an interesting future prospect for our multienemy experimental approach.

The cost of phage resistance is known to be dependent on several factors, such as the genetic background of the phage, the number of the phage strains during the infection, and the environmental conditions (Bohannan and Lenski 2000; Lennon et al. 2007; Lopez-Pascua and Buckling 2008; Koskella et al. 2012). For example, in low nutrient environment such as soil and pond water, the costs for phage resistance are likely to be high (Lopez-Pascua and Buckling 2008; Gomez and Buckling 2011). Thus, the fitness costs constraining bacterial growth could to explain the small but persisting long-term negative effect on free-water phase biomass of the bacteriophages in our experiment.

Both amoeba and ciliate population sizes slowly declined during the experiment. Similar results were found in previous studies with similar predator-prey system, and the decline could attribute to the evolutionary increase in prey defense, while there is little evidence that the predator coevolved (Friman et al. 2008; Friman and Laakso 2011). Alternatively, the culture conditions could have been deteriorating due to an unknown factor in the experiment settings, resulting in the decline of amoeba and ciliate populations. However, unlike with the phages, the potential evolution of defense is not associated markedly to the ability of the predators to keep the bacterial populations in check. In our experiment, we also found that ciliate population size fluctuated more in the multienemy system, suggesting in nature things might be more complicated due to the interactions of different bacterial consumers (Fig.4A). The increased fluctuations in our case probably resulted from the declined population size of the amoeba, due to strong competition by the ciliates. Moreover, it has been demonstrated that the host-parasite coevolution proceeds differently in the presence of predator than without it (Friman and Buckling 2013), and there may thus be also other eco-evolutionary reasons for the observed fluctuations.

To conclude, our results suggest that the top-down effects on bacterial prey depend heavily on the feeding mode of the enemy. Ciliates were very efficient open-water predators, whereas the amoeba was able to reduce bacterial biofilm and also the open-water biomass. This could lead to partial and asymmetric overlap in resource use and possibly to the decline of amoeba and to the observed short-term effect on ciliate population dynamics. We also found that while the bacteriophages were super-efficient parasites in the short-term, the long-term effects of the bacteriophage were negligible. This result arose from extremely rapid appearance of phage resistance and weak ability of the bacteriophages to counteract it. Our results highlight that although data from the single-enemy experiments with bacteria and their predators or their parasites are valuable, they may not be able to reflect the processes occurring in multienemy food webs due to the interactions between the enemies. Moreover, the rapid evolutionary dynamics between the enemy and the host can radically affect the relative importance of predatory protozoa versus the parasitic phages as regulators of the bacterial abundance and spatial distribution.

From the viewpoint of biological control of bacterial pathogens, our results highlight that a single lytic bacteriophage can be effective in the short-term, whereas ciliates and amoeba have more persistent effects on the bacteria. With this said, we suggest that bacteriophages and protozoans together could offer a novel option for sustainable and safe antimicrobial therapy of environmentally growing bacteria. An example of such bacterium with major implications in aquaculture is the fish pathogen *Flavobacterium columnare*, the causative agent of columnaris disease, resulting in substantial financial losses in fish farming annually (for a review see Declercq et al. 2013).
